# Evaluating the translational potential of progesterone treatment following transient cerebral ischaemia in male mice

**DOI:** 10.1186/s12868-014-0131-5

**Published:** 2014-11-29

**Authors:** Raymond Wong, Claire L Gibson, David A Kendall, Philip MW Bath

**Affiliations:** Division of Stroke, University of Nottingham, Clinical Sciences Building, City Hospital Campus, Hucknall Road, Nottingham, NG5 1 PB UK; School of Psychology, University of Leicester, Henry Wellcome Building, Leicester, LE1 9HN UK; School of Biomedical Sciences, University of Nottingham, Medical School, Queen’s Medical Centre, Nottingham, NG7 2UH UK

**Keywords:** Progesterone, Stroke, Mini-pump, Co-morbid, Functional, Mouse

## Abstract

**Background:**

Progesterone is neuroprotective in numerous preclinical CNS injury models including cerebral ischaemia. The aim of this study was two-fold; firstly, we aimed to determine whether progesterone delivery via osmotic mini-pump would confer neuroprotective effects and whether such neuroprotection could be produced in co-morbid animals.

**Results:**

Animals underwent transient middle cerebral artery occlusion. At the onset of reperfusion, mice were injected intraperitoneally with progesterone (8 mg/kg in dimethylsulfoxide). Adult and aged C57 Bl/6 mice were dosed additionally with subcutaneous infusion (1.0 μl/h of a 50 mg/ml progesterone solution) via implanted osmotic minipumps. Mice were allowed to survive for up to 7 days post-ischaemia and assessed for general well-being (mass loss and survival), neurological score, foot fault and t-maze performance. Progesterone reduced neurological deficit [F_(1,2)_ = 5.38, *P* = 0.027] and number of contralateral foot-faults [F_(1,2)_ = 7.36, *P* = 0.0108] in adult, but not aged animals, following ischaemia. In hypertensive animals, progesterone treatment lowered neurological deficit [F_(1,6)_ = 18.31, *P* = 0.0001], reduced contralateral/ipsilateral alternation ratio % [F_(1,2)_ = 17.05, *P* = 0.0006] and time taken to complete trials [F_(1,2)_ = 15.92, *P* = 0.0009] for t-maze.

**Conclusion:**

Post-ischemic progesterone administration via mini-pump delivery is effective in conferring functional improvement in a transient MCAO model in adult mice. Preliminary data suggests such a treatment regimen was not effective in producing a protective effect in aged mice. However, in hypertensive mice, who received post-ischemic progesterone intraperitoneally at the onset of reperfusion had better functional outcomes than control hypertensive mice.

## Background

Stroke is the third leading cause of death, after heart disease and cancer, in the UK and the leading cause of adult disability [1,2]. Few effective pharmacological treatments currently exist for acute ischaemic stroke. Although tissue plasminogen activator is effective, its use is limited by its narrow treatment window [3]. Thus, a main focus of stroke research is the development of novel neuroprotective strategies to reduce the progression of neuronal damage.

Pre-clinical studies have shown progesterone to be neuroprotective following cerebral ischaemia [4-13]. However, these pre-clinical stroke studies have tended to administer it via repeated intraperitoneal (i.p.) injections [14]. Although, a couple of studies have administered progesterone subcutaneously via slow release pellets this has only been the case where treatment commenced prior to the onset of stroke [15,16]. Continuous post-injury progesterone administration has been shown to produce enhanced benefit in traumatic brain injury (TBI) as compared to repeated injections in a rat model after neuronal damage [17]. Whether this benefit of continued post-ischemic administration is also true in a model of stroke has not been demonstrated previously.

In addition, the majority of previously published studies investigating the potential protective effects of progesterone have focused on young, healthy adult animals. However, co-morbidities are common in patients with stroke and have been shown to be negatively correlated with functional outcome [18]. Age is the single most important risk factor for stroke and the incidence of stroke increases with age [19-21]. Another well recognised risk factor for stroke is hypertension and pre-existing hypertension may be present in more than half of stroke patients [22,23]. Hypertension affects both small and large vessels supplying the brain [24], and there is a strong association between hypertension and mortality following stroke [25,26]. The impact of these co-morbidities has not been fully investigated. Therefore, further investigation is required to explore the neuroprotective effectiveness of progesterone in these co-morbid animals.

The aim of the current study was to evaluate if progesterone would be effective in conferring neuroprotection after the onset of experimental stroke when administered via osmotic mini-pump in adult mice. In a prior study from our laboratory, the pharmacokinetics of progesterone in mice using this dosing method were investigated, and found it to be effective in delivering progesterone to the target area of the brain [27]. The delivery of progesterone via osmotic mini-pumps in an experimental stroke model, induced via middle cerebral artery occlusion (MCAO), has not been demonstrated previously; mini-pump delivery could offer a more suitable dosing method with the advantages of reducing peaks and troughs in drug levels, the stress associated with repeated injections, and diminishing levels of release over time as seen with pellet implants. Infusion methods are commonly used clinically to maintain drug concentrations and osmotic mini-pump release of agents at a constant rate mimics this approach. In addition, we conducted some proof-of-principle experiments to determine if progesterone administration, via mini-pump or intraperitoneal administration, was effective in aged or hypertensive mice subjected to focal cerebral ischemia.

## Results

### Effect of progesterone infusion treatment on outcome following MCAO in adult C57 Bl/6 mice

**Figure 1:**
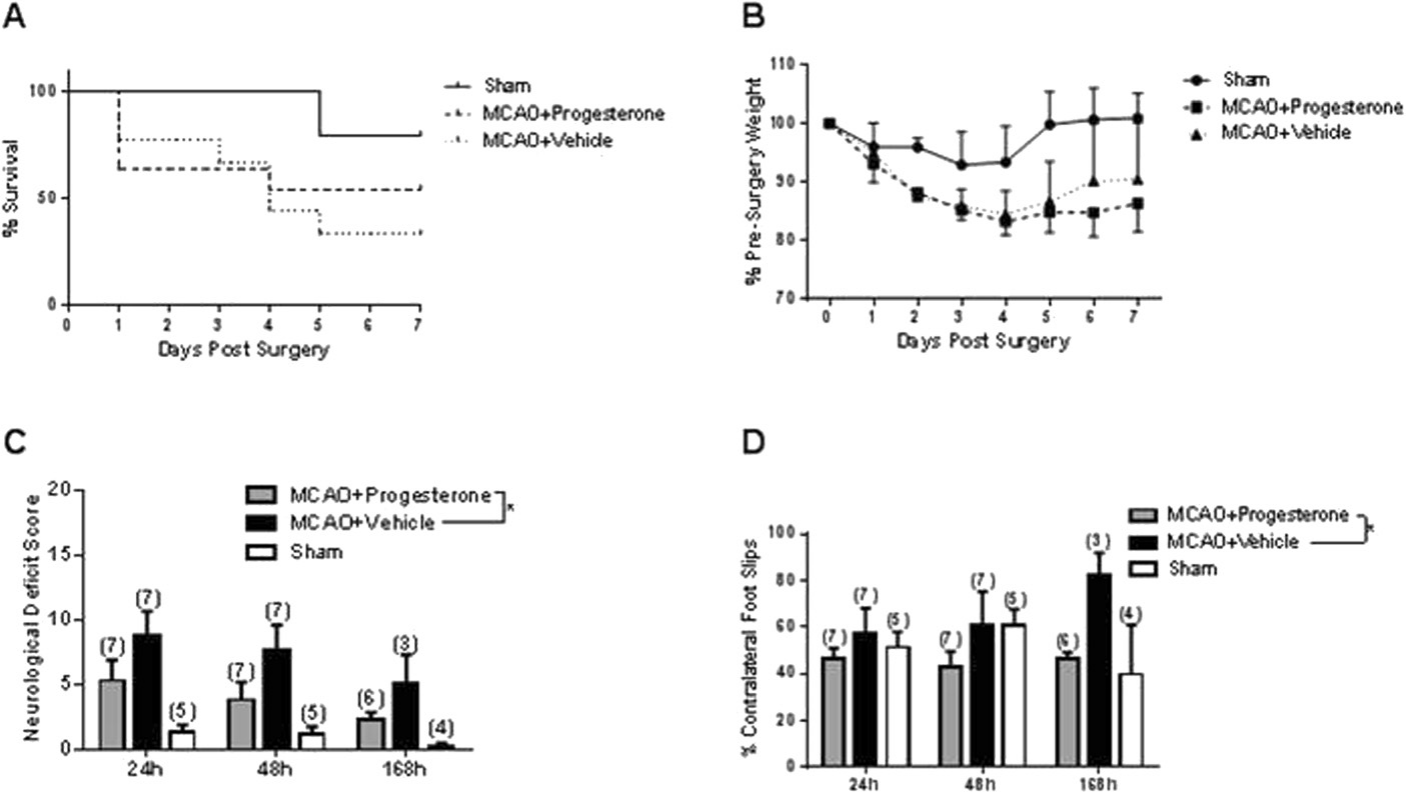
**The effect of progesterone treatment in young adult males.** Comparison of post-surgery survival according to treatment/surgery **(A)**. Mortality data expressed using the Kaplan-Meier curve and analysis using the Mantel-Haenszel log-rank test revealed no significant difference in survival between groups (*P* = 0.3286). In terms of body mass gain following surgery **(B)**, both progesterone and vehicle treated groups gained body mass at significantly slower rate compared to shams (*P* = 0.0004). Neurological deficit scoring **(C)** revealed that MCAO resulted in a significant neurological deficit regardless of whether mice had received progesterone (P = 0.0049) or vehicle (*P* = 0.0002) treatment compared to shams. Progesterone treatment reduced neurological deficit compared to vehicle treatment (*P* = 0.0271). Assessment of motor performance, using the foot-fault test **(D)** revealed that progesterone treatment resulted in significantly fewer contralateral foot-faults compared to vehicle treated animals (*P* = 0.0108). Number of animals per group is shown in parentheses, all data (B-D) are expressed as mean ± SEM, and * = *P* < 0.05.

During the post-operative period a number of animals were sacrificed for welfare reasons; 1 sham-operated mouse (>20% body mass loss), 6 vehicle-treated animals (4 for >20% body mass loss, 2 for barrel rolling), and 5 progesterone-treated animals (1 > 20% body mass loss, 3 for barrel rolling and one found dead the day following surgery). Analysis of survival data revealed no significant differences in survival rate between treatment groups (*P* = 0.3286, Figure 1A). Table 1 shows the number of animals in each experimental group at each time point. All animals lost body mass for the first few days following either MCAO or sham surgery before beginning to gain body mass. Both the progesterone- [F(1,7) = 27.03, *P* = 0.0001] and vehicle-treated [F_(1,7)_ = 13.79, *P* = 0.0004] groups gained body mass at a significantly slower rate compared to shams. There were no significant differences in body body mass gain between the progesterone and vehicle treated groups [F_(1,7)_ = 1.23, *P* = 0.2699] (Figure 1B).

**Table 1:** **Number of mice in each experimental group at each time point**

	**Number of animals per group**						
	**Sham**	**Progesterone**			**Vehicle/Non-treated**		
**Day**	**Adult C57 Bl/6**	**Adult C57 Bl/6**	**Aged C57 Bl/6**	**Hypertensive**	**Adult C57 Bl/6**	**Aged C57 Bl/6**	**Hypertensive**
**1**	5	11	5	5	9	5	4
**2**	5	7		5	7		4
**3**	5	7		5	7		3
**4**	5	7		5	6		3
**5**	4	6		5	4		3
**6**	4	6		5	3		3
**7**	4	6		5	3		3

Body mass for each animal were collected prior to assessing functional outcome or being killed for welfare reasons. Aged mice were not assessed following day 1 as they were excluded for welfare reasons.

Following MCAO, a significant increase in the neurological score, indicative of a neurological deficit, was observed in comparison to sham-operated controls, regardless of whether animals had received progesterone [F_(1,2)_ = 9.34, *P* = 0.005] or vehicle [F_(1,2)_ = 19.12, *P* = 0.0002] treatment. However, progesterone treatment significantly reduced the neurological deficit present following MCAO compared to vehicle treatment [F_(1,2)_ = 5.38, *P* = 0.027 (Figure 1C). Unilateral foot-faults were expressed by the number of contralateral foot-faults as a percentage of the total errors made; a value of 50% represents an equal number of errors made by both sides. For example, on day 1, shams revealed no functional deficit as they made, on average, 51.27 ± 6.62 (mean ± SEM) errors on the contralateral side, whereas, following MCAO, a functional deficit was observed. By day 7, progesterone treatment was found to be beneficial in that it reduced the number of contralateral foot-faults in comparison to vehicle treatment [F_(1,2)_ = 7.36, *P* = 0.0108] (Figure 1D). The T-maze is a test of cognition, including spatial learning and working memory. There was no difference in T-maze performance following either MCAO surgery (in comparison to shams) or by drug treatment. The outcome measures used were: alternation rate %, contralateral/ipsilateral ratio % and time taken to complete trials (Table 2).

**Table 2:** **T-maze data for animals following MCAO**

		**Sham**	**Progesterone**		**Vehicle/Non-treated**	
**Day**	**Outcome**	**Adult C57 Bl/6**	**Adult C57 Bl/6**	**Hypertensive**	**Adult C57 Bl/6**	**Hypertensive**
**5**	**% Alternation**			16.67 ± 7.45		33.33 ± 16.67
	**% Contralateral/Ipsilateral Ratio**			34.29 ± 12.45		61.90 ± 20.76
	**Time Taken (mins)**			3.31 ± 0.27		3.81 ± 0.27
**6**	**% Alternation**			16.67 ± 10.54		11.11 ± 11.11
	**% Contralateral/Ipsilateral Ratio**			25.71 ± 17.14*		95.24 ± 4.76
	**Time Taken (mins)**			3.51 ± 0.27*		5.52 ± 0.80
**7**	**% Alternation**	58.34 ± 4.81	38.89 ± 12.67	26.67 ± 16.33	50.00 ± 9.62	33.33 ± 19.25
	**% Contralateral/Ipsilateral Ratio**	57.14 ± 5.83	54.62 ± 14.03	17.14 ± 10.50	57.14 ± 14.29	71.43 ± 14.29
	**Time Taken (mins)**	7.78 ± 0.94	6.27 ± 0.91	3.84 ± 0.35*	5.59 ± 0.21	5.59 ± 0.76

There was no significant difference between groups in terms of % alternation rate, contralateral/ipsilateral alternation ratio (%), or time taken to complete trials in adult mice (progesterone n = 6, vehicle n = 3, sham n = 4). In hypertensive BPH/2 mice, there was no difference found between progesterone and non-treated groups in t-maze alternation %, but progesterone treated animals had a significantly greater contralateral goal arm preference than non-treated groups (*P* = 0.0006). Data expressed as mean ± SEM (progesterone n = 5, vehicle n = 4).

### Effect of aging on the effectiveness of progesterone treatment following MCAO

**Figure 2:**
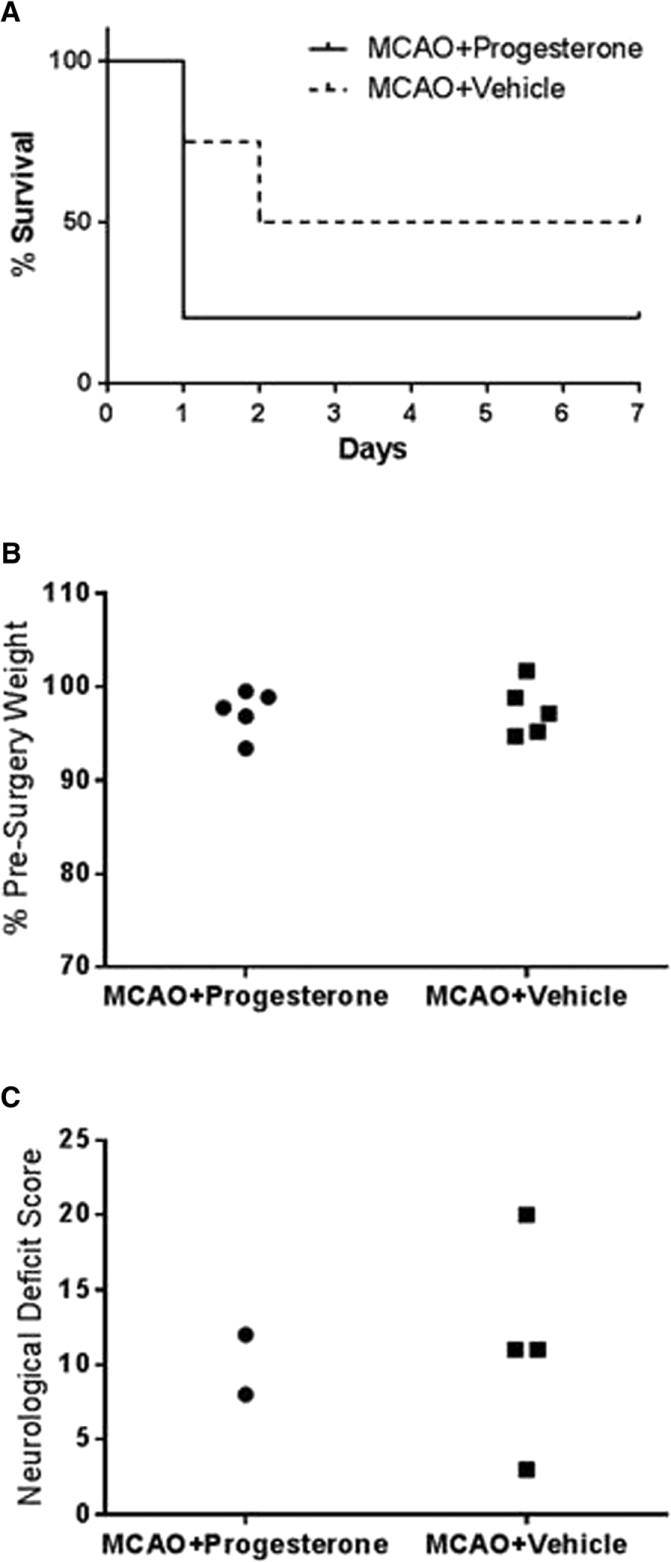
**The effect of progesterone treatment in aged animals.** Comparison of post-surgery survival according to treatment/surgery in aged C57 Bl/6 mice **(A)**. Mortality data expressed using the Kaplan-Meier curve and analysed using the Mantel-Haenszel log-rank test revealed no significant difference between groups in % of surviving animals (*P* = 0.5266). Body mass gain following surgery in aged C57 Bl/6 mice **(B)** at 24 h post-MCAO found no difference in body mass gain between progesterone and vehicle treated animals groups (*P* = 0.8903). Neurological score was assessed in aged C57/Bl6 at 24 h post-MCAO **(C)** no difference was found between treatment groups. Individual data points are shown for body mass and neurological score.

Ten aged C57 Bl/6 mice (five progesterone treated and five vehicle treated) were subjected to 30 minutes of occlusion. In the progesterone treated group only one animal survived to the intended end of study, i.e. day 7 post-MCAO, one was found dead the following day after surgery, and 3 were killed for welfare reasons (barrel rolling). The vehicle-treated group comprised two animals surviving to day 7, one found dead the next day after surgery, and two animals killed for welfare (one for barrel rolling after surgery and another on the same day for inability to recover from surgery). There was no difference between treatment groups for overall survival and body mass loss (Figure 2A and B). Also, no difference between treatment groups for neurological deficit score the day after surgery (progesterone n =2, vehicle n =4) was found (Figure 2C).

### Effect of hypertension on outcome following MCAO and progesterone treatment

**Figure 3:**
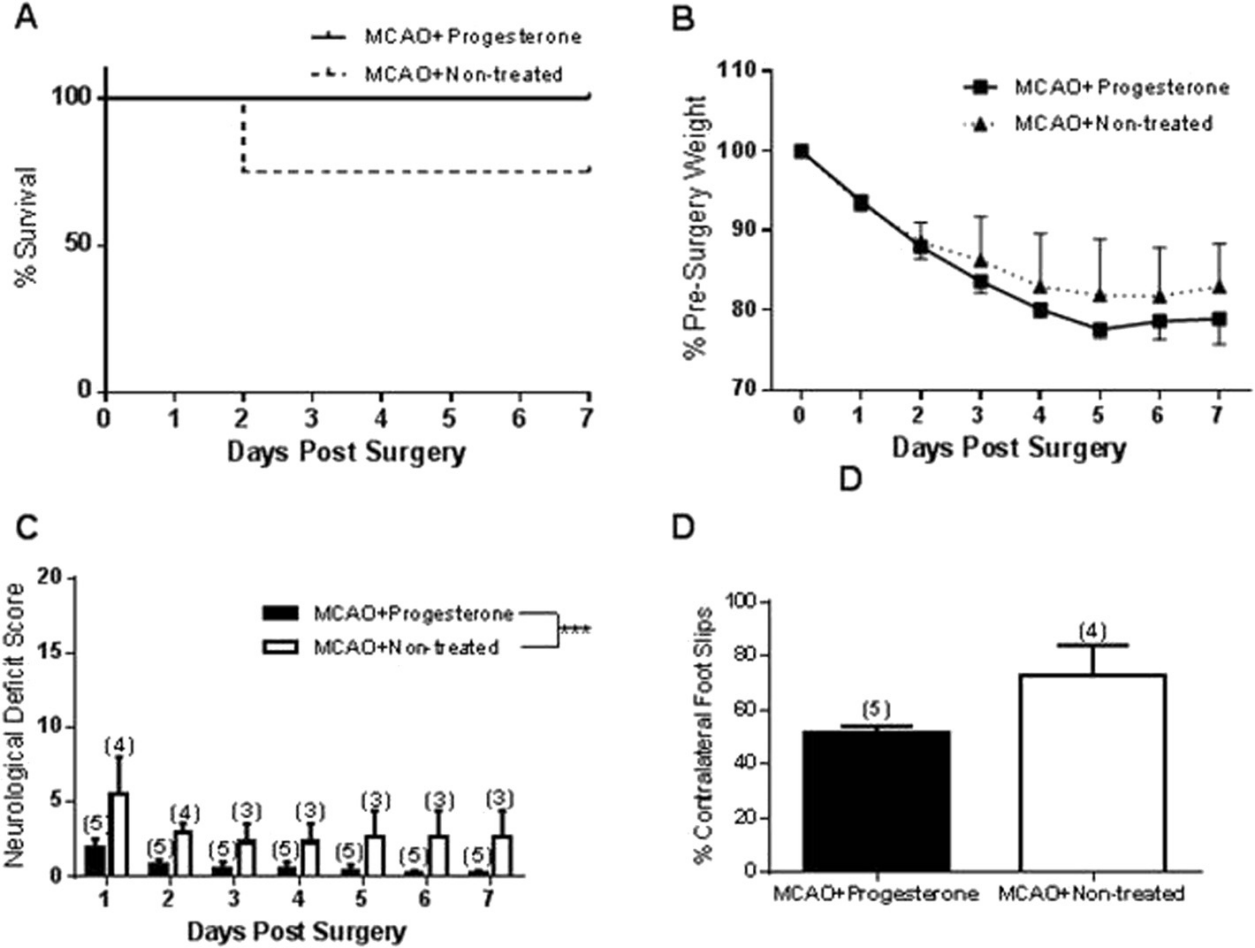
**The effect of hypertension on outcome following MCAO and progesterone treatment.** The percentage of animals survived post-surgery over time **(A)**. Mortality data expressed using the Kaplan-Meier curve and analysed using the Mantel-Haenszel log-rank test showed no significant difference between groups in % of surviving animals. All BPH/2 mice significantly lost body mass after surgery (*P* = 0.0001) but progesterone treatment did not affect the amount of body mass loss **(B)**. Progesterone treatment reduced neurological deficit **(C)** compared to controls (***P = 0.0001). Contralateral foot-faults (%) were assessed in BPH/2 mice on the day following surgery **(D)** and there was no difference found between progesterone and non-treated mice (*P* = 0.0679). Number of animals per group are shown in parentheses and all data **(B-D)** are expressed as mean ± SEM.

All progesterone treated animals (five animals) survived to the end of all functional end-points on day seven and one untreated animal died on day three post-surgery (four animals in untreated group prior surgery). Analysis of survival found no significant difference between progesterone and non-treated animals (Figure 3A). All animals lost body mass following surgery [F_(1,7)_ = 13.53, *P* = <0.0001], but no significant difference was found between groups (Figure 3B). Neurological deficit analysis found progesterone treated mice had significantly lower neurological deficit compared to non-treated animals [F_(1,6)_ = 18.31, *P* = 0.0001] (Figure 3C). There was no difference in contralateral foot slips between groups in the foot fault test (Figure 3D). Analysis of t-maze tasks found no difference in treatment groups in % alternation rate, but did find progesterone treatment to lower contralateral/ipsilateral alternation ratio % [F_(1,2)_ = 17.05, *P* = 0.0006] and reduce the time taken to complete trials for T-maze [F_(1,2)_ = 15.92, *P* = 0.0009] (Table 2). Bonferroni analysis of day 6 shows progesterone treatment significantly increased % left/right ratio (*P* = <0.05*). Non-treated animals took longer to complete t-maze than progesterone treated animals on days 6 and 7 (*P* = 0.0009). Bonferroni post-hoc analysis shows day 6 and 7 to be significant (both *P* = <0.05*).

## Discussion

The current study shows that progesterone treatment, initiated following the onset of MCAO and delivered continually is beneficial in terms of promoting functional recovery in young, adult healthy male mice. In the present study, our outcomes have focused on functional measures, as clinical trials of novel stroke therapies use functional measures as their primary end-point. In humans, the amount of structural damage resulting from the stroke does not always correlate well with functional impairment [28]. Thus, it is important for pre-clinical studies to have functional measures relevant to stroke outcome in patients. A major goal for the treatment of stroke is for patients to be functionally independent and so pre-clinical studies need to reflect the importance of functional outcomes. Restoration of behavioural function needs to be demonstrated in the pre-clinical evaluation of any putative stroke therapy and from the results reported here further longer-term assessments of recovery are warranted.

Previous studies have reported the ability of progesterone to improve functional deficit following MCAO using neurological deficit scales [5,10,15,29-31]. However, these studies have tended to use a much simpler neurological deficit scale which simply grades function by 5 (rats) or 6 (mice) levels ranging from ‘normal’ to ‘severely impaired’ [32,33]. In the current study, we utilised a 28-point neurological deficit score, first developed by Clark *et al*. [34], which we believe is a more sensitive measure of neurological deficit. Functional deficits, as measured by the foot-fault test, found progesterone treatment to be beneficial over time, by reducing the number of contralateral foot-faults in comparison to vehicle treatment. In the current study we chose a relative short occlusion time, in comparison to other studies [6,7,15,16,29], in an attempt to maximize survival following MCAO.

As well as affecting motor behaviours cerebral stroke also commonly disrupts cognitive processes. In rodents, cognitive processes can be assessed by a variety of tasks including the T-maze which is a test of spatial learning and working memory [35]. This behavioural test allows the detection of one-sided spatial neglect and enables both unchallenged locomotion and exploratory motivation to be observed [35]. It’s ability to detect differences in cognitive processing following ischemic stroke have been previously demonstrated [36-40]. For example, animals with ischaemic brain injury have a tendency to turn ipsilaterally towards the damaged side during the t-maze test and display behavioural asymmetry [41,42]. However, in the current study, thirty minutes of MCAO did not appear to affect any of the outcome measures (e.g. alternation rate, goal arm preference) associated with the T-maze in in healthy adult animals. However, in co-morbid animals where the effect of ischemia is exacerbated a deficit in T-maze performance was observed. However, future studies using an extended range of behavioural tests, for example to assess sensorimotor neglect, grip strength etc., are also warranted following progesterone treatment.

Although a number of published studies have reported the protective effects of progesterone treatment for ischemic stroke very few report mortality data. One study reported progesterone treatment to increase survival after ischaemia [7], while others have reported no effect [4,15,29]. In the present study, no difference in survival was found between treatment groups. In terms of welfare measures, body mass can be a useful indicator of general well-being and progesterone has been shown to reduce body mass loss in some studies [7,9,10]. However, in accordance with our study, some have reported that body mass loss, although significant following MCAO, is unaffected by progesterone treatment [5,15,29].

One aim of this study was to determine if sustained progesterone delivery was able to offer neuroprotection following cerebral ischemia as had been previously demonstrated for repeated administrations [6,7,43]. Our previous pharmacokinetic study demonstrated that progesterone delivered via i.p. injection has a very short half-life in both plasma and brain [27] but high progesterone concentrations in the brain can be achieved via mini pump infusion. There have only a few studies, involving progesterone and experimental stroke, which have reported infusion delivery in rats [4] and mice [15]. However, both of these studies initiated progesterone treatment prior to the onset of MCAO whereas in the current study progesterone treatment began following occlusion. The current study demonstrates this method of administration to be beneficial in terms of reducing neurological deficit and improving motor function. Lack of protection in some outcome measures, but not others, may be due to either a dose effect or the length of initial occlusion. Although, increasing the progesterone dose might not necessarily result in greater neuroprotection as some studies have used higher doses of progesterone (10-32 mg/kg via i.p. injection) but found no greater beneficial effect [5,11,44]. Chen *et al.* found 8 mg/kg but not 4 mg/kg or 32 mg/kg to be neuroprotective [5], indicating a relatively narrow dose–response window. Clearly it will be important to determine, in future experiments, whether there is a similar narrow therapeutic window when progesterone is delivered by loading dose and mini-pump infusion, avoiding the peaks and troughs in tissue concentrations produced by repeated injections.

A second purpose of this study was to conduct some proof-of-principle studies in co-morbid animals. In humans, age is a predictor of worse outcome following stroke and this investigation has also shown this to be true in aged animals. Aged animals were very susceptible to MCAO and the majority of aged animals were found dead or were killed for welfare reasons the following day after surgery. Mortality is expected to be higher in older animals compared to younger ones and this has been shown in rats, where mortality immediately post-stroke was found to be low in young animals (6%, 3–4 months) but high in old rats (44%, 18–20 months) [45]. In the current study, the majority of deaths were due to animals being killed for welfare reasons (60%), rather than animals found dead. There was no difference in outcomes between treatment groups in aged animals, although this may be due to lack of statistical power from high mortality in aged animals. Studies have not been previously performed in aged mice that are as old as the ones investigated here (15–16 months of age), which is a phase leaving middle to old age. Middle age, in both mice and rats, is considered to be 10–15 months and old age is considered to be 18–24 months. A limited number of studies have investigated neuroprotection of progesterone in rodents at middle and old age, but most have initiated treatment prior to occlusion and found conflicting results. For example, Toung *et al.* found female rats aged 14–18 months did not benefit from pre-MCAO treatment with progesterone [46], while Alkayed *et al.* using 16 month old female rats found progesterone to be beneficial when administered prior to MCAO [4]. In addition, Gibson *et al.* [29] used 12 month old female mice in which post-MCAO treatment with progesterone was found to be protective in terms of reducing the lesion volume but didn’t improve neurological outcome. In aged (24 months) rats, administration of progesterone post-occlusion did improve both infarct damage and neurological function [30]. Studies of progesterone and experimental stroke involving aged animals are limited, particularly for the dosing of animals after the start of occlusion.

In terms of co-morbidities hypertension is also an important risk factor for ischemic stroke and thought to be present in more than half of stroke patients [22,23]. There is a strong association between hypertension and mortality following stroke [25,26], thus we conducted some preliminary experiments in hypertensive animals. As the outcome in hypertensive mice was expected to be worse we chose a protocol believed to reduce the impact on animal welfare which included a shorter duration time and a one-off intraperitoneal administration of progesterone. This is supported by the fact that hypertensive animsl did display a high percentage loss of body mass in comparison to other animal groups although only showed a relative low neurological deficit in response to the stroke. Progesterone treatment in hypertensive animals was found to be beneficial in reducing neurological deficit, which concurs with work found in hypertensive rats [16]. Although the foot-fault and T-maze tests did not show any significant difference following progesterone treatment in hypertensive mice progesterone treated animals had a preference to turn towards the contralateral side whereas the prediction would be that animals with ischaemic brain injury have a tendency to turn ipsilaterally towards the damaged side during t-maze and other tests of behavioural asymmetry [41,42]. Care must be taken with these findings in hypertensive animals, as our comparison group did not receive vehicle. Progesterone was dissolved in DMSO, which has been shown to have neuroprotective properties [47]. Hence, it cannot be confirmed on the level neuroprotection is attributed to progesterone or DMSO and so further investigation is required to determine this. In addition, alternative mouse models of hypertension, e.g. rennin-dependent, may be worth investigating in comparison to the BPH/2 mice used here.

Studies investigating the neuroprotective potential of drugs in adult healthy animals in experimental stroke do not represent the demographic of patients, which frequently have co-morbidities [18]. Therefore, is important to utilise co-morbid animals in the research of experimental stroke. Also, the presence of co-morbidities, might attenuate neuroprotective effects in some drugs, as seen with hypertension and the experimental neuroprotective agent NXY-059 [48]. In this current preliminary study, only the risk factors of age and hypertension were investigated but other significant risk factors of stroke that were not investigated here include; obesity [49], diabetes [50] and gender [51], which are also overlooked in experimental stroke studies. Also, this study has failed to take into account the combination of risk factors such as age and gender. It would be useful to consider, that whilst pre-menopausal females represent the population group with the lowest risk of stroke, post-menopausal females represent the group at higher risk [51]. Therefore, further investigation is needed in aged female animals along with long-term functional/pathological assessment of outcome which will also permit the study of the effect of neuronal plasticity on recovery.

## Conclusions

Progesterone treatment, initiated post-ischemia, was effective on conferring functional improvement in healthy, adult mice. In addition, such protective effects were observed when delivery of progesterone was sustained thus negating the requirement for repeated injections which results in peaks and troughs of progesterone levels. Any future clinical trials evaluating neuroprotection of progesterone in stroke would need to use a method of dosing that achieved a steady state concentration lasting several days, e.g. infusion or long-acting vaginal/rectal administration. Aged animals have an increased sensitivity to MCAO and did not display, in the outcomes measured here, any benefit from progesterone treatment. Hypertensive BPH/2 mice are a potential hypertensive model and had better functional outcomes after treatment with intraperitoneally administered progesterone, compared to non-treated hypertensive animals.

## Methods

### Animals

Experiments were carried out in accordance with the UK Animals (Scientific Procedures) Act, 1986 (Project License 40/3207) and were approved by the University of Nottingham ethics review process. Twenty five adult male C57 Bl/6 mice (15–25 weeks) were used, weighing between 25.9 and 41.2 g at the time of injection. The aged group was comprised of 15 aged male C57 Bl/6 mice (61–65 weeks), weighing between 34.10 and 46.5 g at the time of injection. Nine male BPH2/2 (hypertensive) mice (28–37 weeks) were used, weighing between 21.9 and 34.8 g at the time of occlusion (5 progesterone treated and 4 non-treated animals). No differences were seen in body weight between treatment groups. All mice were assigned a code by a non-experimenter and assigned randomly to different treatment groups. During all surgical procedures and behavioural tests the experimenter was blinded to treatment.

### Focal cerebral ischaemia

Anaesthesia was induced by inhalation of 4% isoflurane (in 100% Oxygen) and maintained by inhalation of 1.5% isoflurane. Body temperature was monitored throughout surgery (via rectal probe) and maintained at 37°C ± 0.5°C using a heating blanket (Harvard Apparatus, Edenbridge, Kent, UK). Laser Doppler flowmetry (Moor Instruments, Sussex, UK) was used to monitor cerebral blood flow. Focal cerebral ischaemia was induced by middle cerebral artery occlusion (MCAO) as previously described [6]. Briefly, a small incision was made in the skin overlying the temporalis muscle and a 0.7-mm flexible laser-Doppler probe (model P10) was positioned on the superior part of the temporal bone (6 mm lateral and 2 mm posterior from bregma), secured by superglue (Loctite). A midline incision was made on the ventral surface of the neck and the right common carotid artery isolated and ligated. The internal carotid artery and the pterygopalatine artery were temporarily occluded using a microvascular clip (Ohwa Tsusho Co., Tokyo, Japan). A nylon filament (Drennan, Stridgewarer, UK), exposed to heat to give a diameter of 180 μm, was introduced into the internal carotid artery via an incision in the common carotid artery. The filament was advanced approximately 10 mm distal to the carotid bifurcation, beyond the origin of the middle cerebral artery. Relative cerebral blood flow (CBF) was monitored for the first 5 minutes following MCAO, during which time relative CBF had to reduce to at least 70% of pre-ischaemic values for inclusion, before mice were allowed to return to their home cage. After 15 (hypertensive animals) to 30 minutes of occlusion (adult and aged animals), mice were re-anesthetised and the occluding filament was withdrawn back into the common carotid artery to allow reperfusion to take place. At this stage drugs were administered and osmotic mini-pumps implanted (see drug treatment below). Relative cerebral blood flow was monitored for an additional 5 minutes before the wound was sutured and mice were allowed to recover from the anaesthesia at 22°C. The relative cerebral blood flow had to rise to at least 50% of pre-ischaemic values for mice to be included in the study and subject to further analyses (and independent of which treatment they received). Sham-operated mice underwent the same surgical procedure, except that the filament was not advanced far enough to occlude the middle cerebral artery and they received empty mini-pumps.

### Drug treatment

Following random allocation, mice were injected intraperitoneally, at the onset of reperfusion, with a ‘loading dose’ of either progesterone (USP grade, 8 mg/kg in a solution of 16 mg/ml in 100% DMSO, Sigma, St Louise, MO, U.S.A) or vehicle (100% DMSO). Mini-pumps, with an infusion rate of 1.0 μl/hr and a reservoir with up to 3 days delivery capacity (Alzet 1003D), were loaded with progesterone solution (50 mg/ml progesterone dissolved at 37°C in 100% DMSO only) or 100% DMSO (placebo) as previously described [27]. The pumps were submerged in 0.9% sterile saline solution at 37°C over night in order to prime them so that progesterone was delivered immediately after implantation. Mini-pumps were implanted subcutaneously in the back immediately following the onset of reperfusion and administration of the initial loading dose (progesterone or vehicle) as stated above. Hypertensive BPH/2 mice only received the intraperitoneal injection of the ‘loading dose’ of progesterone at the onset of reperfusion.

### Assessment of general well-being

After surgery, mice were weighed every day for 7 days as an indication of their general well-being [7]. Body mass data are presented as a percent change compared with body mass on the day of surgery. Survival rates of animal groups are also presented as % compared to the number of animals undergoing surgery.

### Neurological deficits

Animals were scored neurologically for focal deficits with the use of a 28-point neurological scoring system based from Clark *et al.* 1998 [34]. This 28-point scale awards a score of 0–4 (0 = normal, 4 = most severely affected) on 7 different characteristics by a variety of assessment methods: (i) body symmetry – assessed by observation on open bench, (ii) gait – assessed by observation on open bench, (iii) climbing – assessed by observing gripping at 45°, (iv) circling behaviour – assessed by observation on open bench, (v) front limb symmetry – assessed via tail suspension, (vi) compulsory circling – assessed by allowing front limbs to be placed on bench during tail suspension, and, (vii) whisker response – assessed via light touch from behind.

### Foot fault

As previously described [7] at 24 h, 48 h and 7 days after MCAO or sham surgery, mice were placed on an elevated grid surface (30 × 35 × 31 cm) with grid openings of 2.5 cm^2^. During locomotion on the grid, the number of foot faults made by the ipsilateral and contralateral limbs was counted. Ipsilateral refers to those limbs on the same side of the body i.e. right, as which the vessel occlusion took place whereas contralateral limbs refers to those on the side of the body opposite to where vessel occlusion occurred. Tests consisted of 3 trials of 1 minute each with an interval of 1 minute between trials. The foot faults are expressed as the number of errors made by the contralateral side limbs in % of the total errors made.

### T-maze

The T-maze consists of 3 arms made of grey Perspex, each 41.5 cm long and 6 cm wide, surrounded by walls of transparent Perspex (15 cm high). The start box (6 cm x 7.5 cm) is located at the bottom of the central arm. The start box and the entrance of each arm can be closed by vertical sliding doors. Mice were subjected to a series of 1 habituation period and 7 trials separated by 5 second intervals. The habituation period consists of 1 minute during which the mouse was left to explore the entire maze and allowed to return to the start position. At the start of a trial the door is opened and the mice have the option of entering either the left or right arm. The mice were then left in the arm of choice for 5 seconds and recorded before being allowed to return to the start box. If they did not freely return to the start box or remained stationary for 15 seconds, they are gently nudged to avoid stress of handling. Alternation rate (% alternation), which assesses the willingness of mice to explore a new environment, i.e. whether they prefer to visit a new arm of the maze rather than a familiar arm, along with contralateral/ipsilateral (% contralateral ipsilateral ratio) of arm choice relative to stroke hemisphere were measured. The total time was recorded on how long the animals took to complete the 7 trials. T-maze was conducted on day 7 after surgery for adult and aged mice and days 5, 6 and 7 for hypertensive mice.

### Statistical analysis

All data are expressed as means ± standard error of the means (SEM). Survival data was analysed by Kaplan-Meier curve and Mantel-Haenszel log-rank test to identify differences. All other experiments conducted over a period of days (body mass, neurological score and foot fault) were analysed by two-way analysis of variance (ANOVA) for differences according to time and treatment. The data from the T-maze was analysed using one-way ANOVA to identify differences according to treatment. Table 1 shows the number of animals in each experimental group at each time point. *Post hoc* analyses were carried out using Bonferroni tests. Data analysis was conducted using GraphPad Prism Version 5.0 for windows (GraphPad Software). The criterion for statistical significance was *P* <0.05.

### Compliance with ethics requirements

All institutional and national guidelines for the care and use of laboratory animals were followed.
